# Analysis of Intervertebral Disc Degeneration Induced by Endplate Drilling or Needle Puncture in Complement C6-Sufficient and C6-Deficient Rabbits

**DOI:** 10.3390/biomedicines12081692

**Published:** 2024-07-30

**Authors:** Amelie Kuhn, Markus Huber-Lang, Sebastian Weckbach, Jana Riegger, Graciosa Q. Teixeira, Volker Rasche, Jörg Fiedler, Cornelia Neidlinger-Wilke, Rolf E. Brenner

**Affiliations:** 1 Division for Biochemistry of Joint and Connective Tissue Diseases, Department of Orthopedics, Ulm University, 89081 Ulm, Germany; amelie.kuhn@uni-ulm.de (A.K.); jana.riegger@uni-ulm.de (J.R.); joerg.fiedler@uni-ulm.de (J.F.); 2 Institute of Clinical and Experimental Trauma-Immunology, University Hospital Ulm, Ulm University, 89081 Ulm, Germany; markus.huber-lang@uni-ulm.de; 3 Department of Orthopedic Surgery, RKU, Ulm University, 89081 Ulm, Germany; sebastian.weckbach@gmx.de; 4 Institute of Orthopedic Research and Biomechanics, Trauma Research Centre, Ulm University, 89081 Ulm, Germany; graciosa.teixeira@uni-ulm.de (G.Q.T.); cornelia.neidlinger-wilke@uni-ulm.de (C.N.-W.); 5 Department of Internal Medicine II, Ulm University, 89081 Ulm, Germany; volker.rasche@uni-ulm.de; 6 Core Facility Small Animal Imaging (CF-SANI), Ulm University, 89081 Ulm, Germany

**Keywords:** disc degeneration, rabbit, EP drilling, needle puncture, MRI analysis, DHI analysis, complement, C6 deficiency

## Abstract

Previous studies indicate an implication of the terminal complement complex (TCC) in disc degeneration (DD). To investigate the functional role of TCC in trauma-induced DD in vivo, the model of endplate (EP) drilling was first applied in rabbits using a C6-deficient rabbit strain in which no TCC formation was possible. In parallel the model of needle puncture was investigated. Using a minimally invasive surgical intervention, lumbar rabbit intervertebral discs (IVDs) were treated with EP drilling or needle puncture. Degenerative effects of both surgical interventions were assessed by Pfirrmann grading and T2 quantification of the IVDs based on high-resolution MRI (11.7 T), as well as radiographic determination of disc height index. Pfirrmann grading indicated significant degenerative effects after EP drilling. Contrary to our assumption, no evidence was found that the absence of TCC formation in C6-deficient rabbits reduces the development of DD compared to C6-sufficient animals. EP drilling was proven to be suitable for application in rabbits. However, results of the present study do not provide clear evidence of a central functional role of TCC within DD and suggest that TCC deposition in DD patients may be primarily considered as a marker of complement activation during DD progression.

## 1. Introduction

Lower back pain is one of the most frequent and cost-intensive health problems of Western society, and it is often associated with degenerative changes of the intervertebral disc (IVD) [1,2]. Besides genetic factors, age, chronic mechanical stress, and smoking, trauma represents an important risk factor for IVD degeneration (DD) [3]. Especially, injuries of the endplate (EP) can trigger post-traumatic degeneration of the adjacent IVD [4,5,6,7].

DD is associated with an imbalanced nutrition of the tissue, increase in proinflammatory factors, enhanced matrix degradation by an induction of catabolic enzymes [8,9], and reduced proteoglycan content of the IVD, leading to a decreased water-binding capacity of the tissue [10,11,12]. In turn, this results in a diminished capability to maintain physical pressure and causes a consequent reduction in disc height [10,11,12].

In degenerated human IVDs, an enhanced deposition of the terminal complement complex (TCC), the downstream activation product of the complement cascade consisting of C5b, C6, C7, C8, and C9, which can induce cellular signaling, inflammation, cell death, and inflammatory response, was previously described [13]. In more detail, we could even demonstrate that TCC deposition observed in clinical IVD samples positively correlates with the grade of degeneration indicated by the Pfirrmann score [14]. Furthermore, our investigations demonstrated that complement activation induces the gene expression of catabolic enzymes in annulus fibrosus (AF) cells; therefore, this strengthened the hypothesis of a possible influence of a complement activation in the progress of DD [15]. Moreover, a central role of TCC in the development of osteoarthritis, a degenerative joint disease that is accompanied by inflammation, was previously described [16,17]. However, it is not yet clarified if the TCC itself or rather the anaphylatoxins, such as C3a and C5a, both of which are generated upstream in the complement activation cascade [18], are crucial for the functional implication of complement activation in DD pathogenesis [19].

To investigate the underlying mechanisms of DD development, a variety of animal models based on different surgical interventions have been used [20,21], all of which lack unrestricted transferability to the pathogenetic conditions of human DD. For example, by applying a scalpel incision into the anterior AF in the stab injury model [22], degenerative effects in the NP are induced by the herniation of NP material; however, alterations of the AF are predominantly induced only at the site of the injury [23]. However, in IVDs of DD patients, degenerative change of the AF is evenly widespread instead of a local restriction to a single site. 

To evaluate the role of TCC deposition in trauma-induced initiation and progression of DD in vivo, a DD model in which TCC formation is not possible would be desirable. A previously described rabbit strain with a C6 deficiency based on an inherited lack of functional C6 protein fulfills these requirements [24,25]. As the binding of the central complement component C6 to C5b is the initial step of TCC formation, the lack of functional C6 blocks all subsequent assembly steps of TCC formation [26]. 

One of the most commonly used models for the induction of mild IVD degeneration in rabbits is the method of needle puncture, by which a stab wound in the AF is created, which subsequently induces degenerative changes of IVD tissue [27,28]. Beyond that, Holm et al. [23] established a model of EP drilling in domestic pigs, which represents a surgical intervention that fairly mimics the conditions of a human EP lesion leading to DD. It is based on a drilling through the vertebral body and the EP into the nucleus pulposus (NP) of a lumbar IVD, which induces degeneration of the IVD [23]. In contrast to one-sided degenerative effects induced by a stab wound that are restricted to the injured side of the AF, EP injury initiates degeneration from the central part of the IVD, thus inducing a more symmetric and widespread disruption of the AF [23]. Compared to needle puncture, EP drilling is expected to be associated with a greater local exposure of complement factors from the blood. Furthermore, several proteases of the blood coagulation system have been shown to be able to activate the complement cascade independently of the common complement pathways [29].

Therefore, in the present study, the model of EP drilling was applied for the first time in rabbits, besides needle puncture of the IVD, as a commonly used method for the development of mild DD. In order to reduce the burden of the procedure as well as the risk of infection, surgical interventions were performed as minimally invasive procedures. Degenerative effects of applied interventional methods in C6-sufficient (C6^+/−^) and C6-deficient (C6^−/−^) animals were evaluated via MRI analysis of the IVDs and radiographic assessment of the disc height, respectively, to investigate if TCC deposition has an impact on DD development. In every study animal, one lumbar IVD was punctured using a 20 G needle and a second lumbar IVD was treated with EP drilling. The surgical interventions were performed in an alternating arrangement from L1/2 to L4/5, enabling untreated segments of the respective IVD level to serve as controls without the requirement of additional animals.

## 2. Materials and Methods

### 2.1. Animals

Animal experiments were performed according to the European Union Directive 2010/63/EU and the international regulations for the care and use of laboratory animals and were approved by the local ethical committee (Regierungspraesidium, Tuebingen, Germany, reg. number 1318). In the animal experiment, C6-deficient (C6^−/−^) rabbits originally descending from a rabbit with a C6 deficiency obtained from Prof. Dr. Sucharit Bhakdi, Institute of Medical Microbiology and Hygiene, University of Mainz, Germany, were used [30]. The C6 deficiency of respective animals is based on a naturally occurring mutation in *C6*, resulting in a loss of functional C6 protein [25]. Overall, 17 adult rabbits were used in this study (*n* = 8 C6-deficient (homozygous; C6^−/−^) and *n* = 9 C6-sufficient (heterozygous animals of the same breed; C6^+/−^); C6^+/−^: 38.8 ± 2.9 weeks old, 3.9 ± 0.4 kg; C6^−/−^: 38.5 ± 2.6 weeks old, 3.7 ± 0.2 kg; for detailed information, see Supplementary Table S1). The genotype of the rabbits was determined on a functional level by an erythrocytes lysis assay and confirmed by PCR-based analysis of the mutation in each animal, as previously described [25] (Supplementary Figure S1). 

In accordance with the 3R rule (Replacement, Reduction, Refinement), animals of both genders obtained from the breeding were used in a balanced ratio. Under further consideration of the 3R guideline, C6^+/−^ rabbits served as the control group. Serum of C6^+/−^ rabbits exhibited identical complement-associated hemolytic activity compared to New Zealand white rabbits (C6^+/+^), with C6^+/−^ rabbits proving to be suitable controls in previous animal studies using this animal model [25,30]. No general health problems were observed in C6^−/−^ rabbits.

### 2.2. Surgical Procedure

The rabbits were anesthetized with initial intravenous injection of Xylazine (0.5 mg/kg; BAYER, Zurich, Switzerland) and Ketamin (7.5 mg/kg; WDT, Garbsen, Germany), as well as subsequent intravenous dosing, as required. Their fur was shaved in the lower area of the back, and the animals were placed in a strictly lateral position laying on their left side. 

Regarding a precise needle application based on the IVD dimensions of the present rabbit strain and considering that noticeable degenerative changes were described for applying a needle diameter of 21 G [28], one of the lumbar IVDs L1/2 to L4/5 was punctured with a 20 G needle based on the procedure described by Kwon [27]. Another lumbar IVD was treated with EP drilling using a 1.5 mm drill modified for rabbits based on the principles, as described previously for application in domestic pigs [23]. Thereby, the respective treatments were applied to different IVD levels per animal in an alternating arrangement of treatment and controls, including all participating IVD levels equally (compare Table 1). 

**Figure 1:**
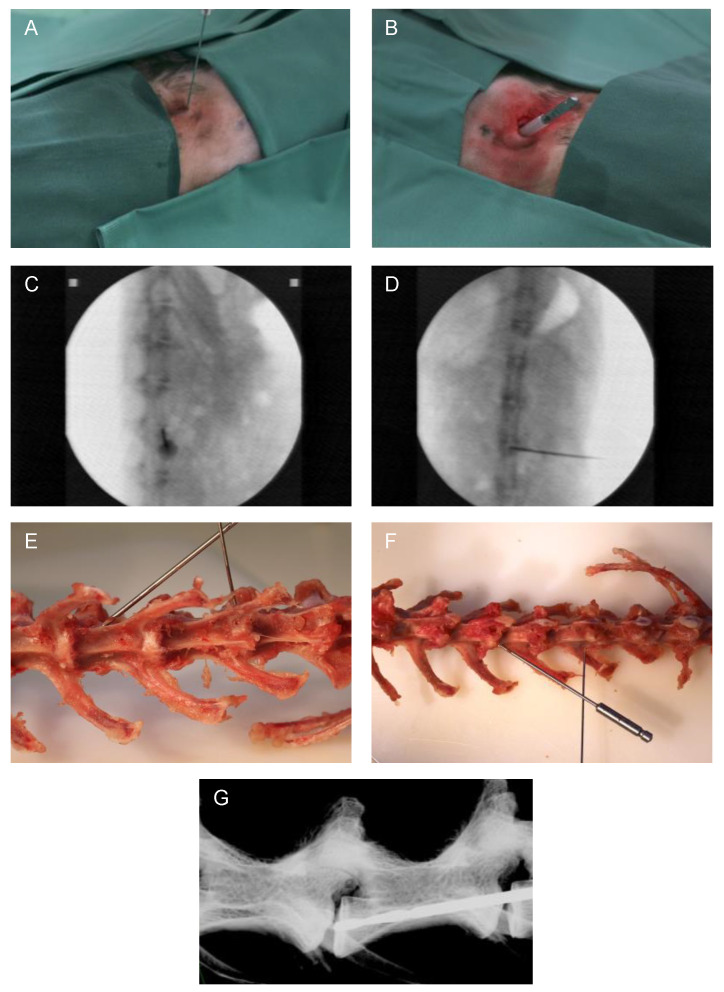
Representative images of the surgical method. (**A**) Minimally invasive needle puncture and (**B**) EP drilling of a lumbar rabbit IVD. Intraoperative validation of the needle position by X-ray in (**C**) sagittal and (**D**) coronal planes (C-arm). Exemplary visualization of needle puncture (L1/2) and EP drilling (L3/4) representing needle and drill position on an explanted rabbit spine in (**E**) ventral and (**F**) dorsal views. (**G**) Display of drill position by X-ray of an explanted spine (Faxitron X-ray system, Tucson, AZ, USA).

The surgical procedure was minimally invasive and carried out under radiographic guidance [27] (C-arm, Exposcop CB7-D, ZIEHM, Nuremberg, Germany) in sagittal view. At the level of the respective IVD to be addressed, a 1 cm skin incision was performed, and the IVD was reached by blunt dissection. For needle puncture (Figure 1A), the needle was transversally inserted into the center of the IVD, thus reaching the NP. Afterwards, the needle was rotated by 180° [27]. Through a second 1 cm incision (and blunt dissection up to the IVD) for EP drilling with a compressed air drill, the drill was positioned into the central part of the EP in caudal direction at a 45° angle (positioning compare Figure 1E–G). For preventing damage of the surrounding tissue, a cut off plastic needle cap was used as a drill sleeve to protect the tissue during minimally invasive EP drilling (Figure 1B). Correct position of the needle or drill was verified in each case by an additional X-ray overview in the coronal view (Figure 1D). Finally, after rinsing the surgical site, the skin incisions were closed with intracutaneous sutures using absorbable suture material.

After surgery, the animals received Buprenorphine (0.03 mg/kg, s.c.; BAYER, Zurich, Switzerland) two times daily for analgesia and Enrofloxazine (7.5 mg/kg, s.c.; WDT, Garbsen, Germany) once daily for antibiotic prophylaxis over three days. Upon animal harvest, animal welfare was continuously monitored (daily in the first week post-op, twice weekly in the second week, and then weekly) and evaluated on the basis of coat care, spontaneous behavior, eyes/gaze, water balance based on examination of skin folds, reaction to touch, body temperature, condition of the surgical wound, movement behavior, food and water intake, and body weight (Supplementary Figure S2). Twelve weeks after surgery, the rabbits were sacrificed with bolt shot and subsequent lethal bleeding from cervical arteries.

### 2.3. Post-Mortem X-ray and MRI Analyses

The lumbar spines of the rabbits were explanted directly after sacrifice. Ex vivo X-ray scans were performed in the sagittal plane (MX-20, Faxitron Bioptics; Tucson, AZ, USA (35 kV, 19 s)), and the disc height index (DHI) was determined according to Masuda et al. [28].

Furthermore, MRI analyses were performed using a 11.7 T MRI scanner (Bruker, Billerica, MA, USA, 72 mm T/R coil, 150^2^ × 750 µm^3^ spatial resolution, 1.05 mm interslice gap, T2-weighted spin echo sequence with echo/repetition time of TE = 18 ms/TR = 2500 ms, Core Facility for Small Animal Imaging, Medical Faculty of Ulm University) to assess the water content of the IVDs, which is related to the proteoglycan content of the tissue [31]. 

Classification of degenerative changes according to Pfirrmann grading [32] was performed by two independent observers based on midsagittal MRI scans of the rabbit IVDs. Corresponding interobserver variability was assessed by means of linear weighted kappa according to Rim [33]. Grading results of both observers varied by a maximum of one grade and had an agreement of 60%. With a kappa value of 0.59, interobserver agreement was determined as “moderate” [33,34].

To further quantify the degenerative changes in lumbar IVDs, T2 values of the IVDs were analyzed. Thereby, the average T2 values of the central three-dimensional IVD region were determined based on T2-weighted MRI scans in sagittal and coronal planes (TE = 15–275 ms, TR = 7200 ms with inter-echo spacing of 15 ms) acquired with similar spatial resolution as reported above. Mean T2 values of individual needle-punctured and EP-drilled IVDs were determined from the central sagittal respective coronal sectional planes (7–9 central sagittal planes, 4–5 central coronal planes) and were normalized to the average T2 values of untreated control IVDs of the corresponding IVD level and genotype, for sagittal and coronal planes separately.

### 2.4. Statistics

Normality of the data was confirmed using the Shapiro–Wilk normality test, and statistical analysis was performed with parametric one-way ANOVA, followed by Sidak’s multiple comparison test, using GraphPad Prism 9.4.2 for Macintosh (GraphPad Software, Inc., La Jolla, CA, USA). For the statistical analysis of two independent groups (C6^+/−^ vs. C6^−/−^), an unpaired *t* test was performed. Statistical significance was considered at *p* < 0.05. 

## 3. Results

Of the 17 animals of this study, 16 rabbits exhibited normal spontaneous behavior directly after awaking from the anesthesia. The animals did not show any signs of pain, movement restrictions, or neurological deficiencies. Detailed aspects continuously monitored throughout the entire course of this study are mentioned in Section 2. One C6^+/−^ rabbit showed post-operative lameness of a hind leg but completely recovered within 48 h. Moreover, during the entire post-operative period, the adult animals did not show significant weight loss, indicating reduced food intake (Supplementary Figure S2).

12 weeks after surgical intervention, explanted lumbar rabbit spines were analyzed using X-ray and 11.7 T MRI to assess degenerative changes of needle-punctured and EP-drilled IVDs.

Unintentionally resulting adjacent segment degeneration affecting untreated IVDs next to needle-punctured or EP-drilled IVDs, which served as untreated control IVDs, was excluded by comparing T2 values and DHI of untreated control IVDs with exemplary investigated IVDs of the respective IVD level from completely untreated C6^+/−^ and C6^−/−^ rabbits.

Because of the anatomic proximity to the ribs, the precise positioning of the drill including the correct applicating angle was very challenging at IVD level L1/2. Strikingly, none of the IVDs of this level treated with EP drilling exhibited any signs of degeneration in the MRI images. Since standardized EP drilling could not be confirmed with certainty at this anatomical level, all IVDs of level L1/2 treated with EP drilling were excluded from further analyses.

### 3.1. Visual Evaluation/Pfirrmann Scoring of MRI Scans Indicates a Clear Degenerative Effect of EP Drilling in IVDs of C6-Sufficient and C6-Deficient Rabbits

**Figure 2:**
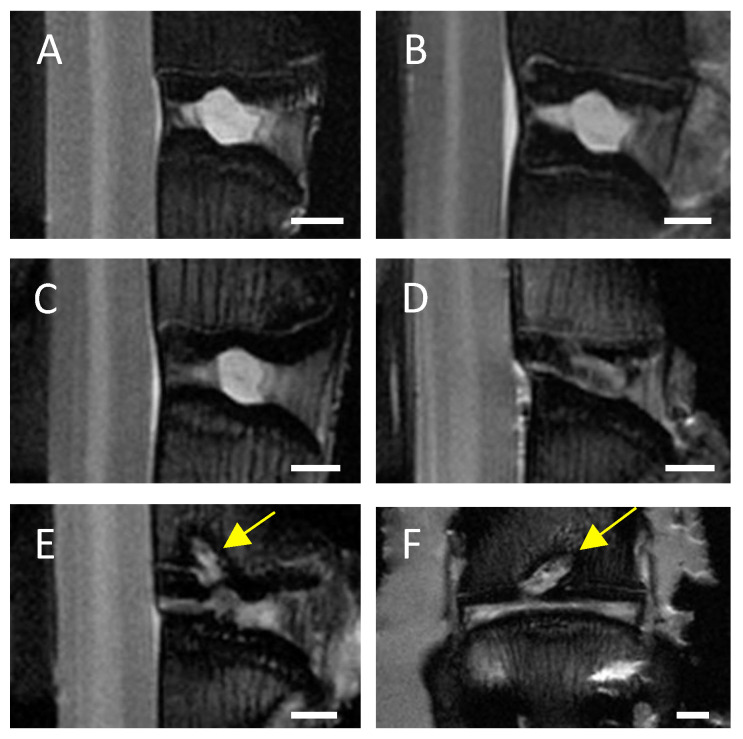
Representative central MRI images. Exemplary midsagittal MRI images of lumbar rabbit IVDs exhibiting (**A**) no signs of degeneration, signs of (**B**) mild degeneration (inhomogeneous IVD structure with slight grey bands), (**C**) clear degeneration (inhomogeneous IVD structure and loss of signal intensity), and (**D**) severe degeneration (grey to black inhomogeneous IVD structure, drastically reduced signal intensity, and unclear distinction between AF and NP). Exemplary (**E**) midsagittal and (**F**) midcoronal MRI scan of an EP-drilled IVD with visible drilling channel (marked by yellow arrows). Scale bar: 2 mm.

In MRI scans of untreated control IVDs and needle-punctured IVDs no or only minimal signs of degeneration, such as an inhomogeneous IVD structure with slight grey bands, were visible in both C6^+/−^ and C6^−/−^ rabbits (Figure 2A,B). Based on visual evaluation of the MRI scans, EP-drilled IVDs generally appeared more degenerated; 5 of 13 analyzed IVDs treated with EP drilling were clearly degenerated (C6^+/−^ *n* = 3 of 7, C6^−/−^ *n* = 2 of 6; Figure 2C) and exhibited an inhomogeneous IVD structure with the loss of signal intensity. Another 4 of the 13 analyzed EP-drilled IVDs furthermore exhibited signs of severe degeneration, such as a grey to black inhomogeneous IVD structure with drastically reduced signal intensity and unclear or even lost distinction between AF and NP (C6^+/−^ *n* = 2 of 7, C6^−/−^ *n* = 2 of 6; Figure 2D). In a total of 5 animals, the drilling channel within the adjacent vertebral bodies of the IVDs treated with EP drilling was visible in the MRI images 12 weeks after surgery (C6^+/−^ *n* = 1 of 7 [14%], C6^−/−^ *n* = 4 of 6 [67%]; see Figure 2E,F).

**Figure 3:**
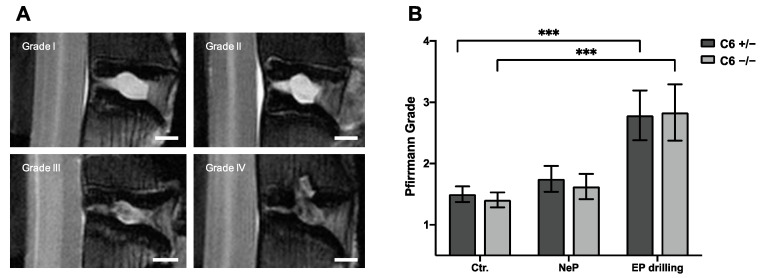
Pfirrmann grading of lumbar rabbit IVDs. (**A**) Exemplary images of rabbit IVDs of Pfirrmann grade I to IV. Scale bar: 2 mm. (**B**) Pfirrmann grades of untreated control IVDs (Ctr.) and IVDs treated with needle puncture (NeP) or EP drilling of C6^+/−^ and C6^−/−^ rabbits. Results are given as mean ± SEM (Ctr.: *n* = 16–18 per group, NeP: *n* = 8 per group, EP drilling: *n* = 6–7 per group). Significant differences are depicted as *** *p* < 0.001; one-way ANOVA.

To classify the degenerative changes observed in lumbar rabbit IVDs, grading according to Pfirrmann et al. [32] was performed based on T2-weighted midsagittal MRI scans. Figure 3B represents the mean results of Pfirrmann grading performed by two independent observers. 

Based on the Pfirrmann grading, no degenerative effect of needle puncture could be observed in C6^+/−^ and C6^−/−^ rabbits (Figure 3B). After EP drilling, the Pfirrmann score of lumbar rabbit IVDs was significantly increased compared to untreated control IVDs (*p* < 0.001), irrespective of the genotype. Exemplary images for the classification to the Pfirrmann grades I to IV are depicted in Figure 3A. None of the lumbar IVDs of the present study were classified as Pfirrmann grade V.

### 3.2. EP Drilling Can Induce a Severe Reduction in T2 Values in IVDs of C6-Sufficient and C6-Deficient Animals

Degenerative changes of treated rabbit IVDs were further analyzed by T2 quantification of T2-weighted MRI scans in sagittal and coronal planes. Thereby, T2 values of needle-punctured or EP-drilled IVDs were normalized to the mean T2 value of untreated control IVDs of the corresponding IVD level for both genotypes, separately.

**Figure 4:**
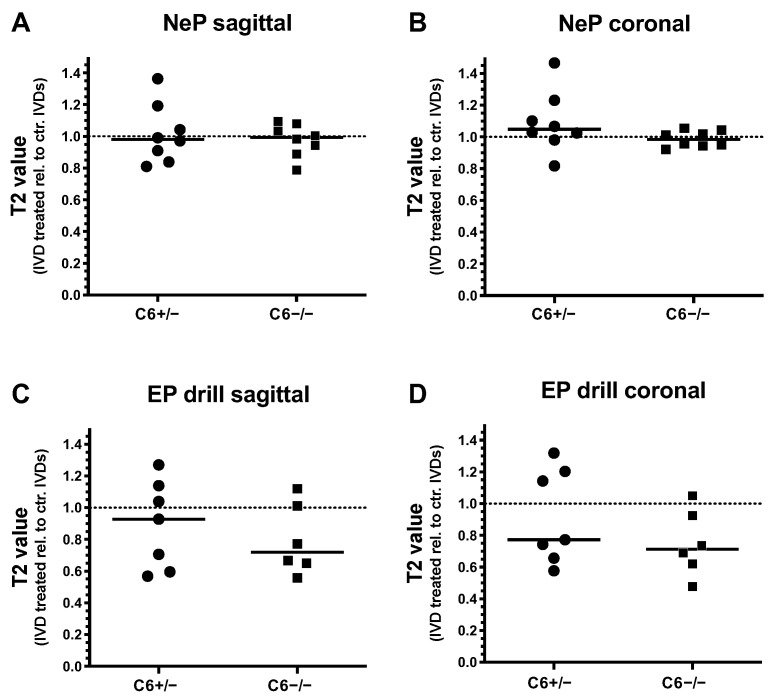
T2 values of needle-punctured (NeP) and EP-drilled (EP drill) lumbar rabbit IVDs relative to untreated control IVDs (dashed lines). T2 values of IVDs treated with NeP (**A**,**B**) and EP drilling (**C**,**D**) are determined from (**A**,**C**) sagittal and (**B**,**D**) coronal T2-weighted MRI scans of lumbar IVDs from C6^+/−^ and C6^−/−^ rabbits. Thereby, T2 values were determined by manually defining the overall IVD area as regions of interest (ROIs). Individual data points represent the mean T2 values of an individual IVD normalized to the average T2 value of untreated control IVDs of the respective IVD level under consideration of the genotype and sectional plane. Relative T2 values are presented as scatter dot plots with lines at median (EP drilled: *n* = 6–7, NeP: *n* = 8 per group). No significant differences were determined between both genotypes; unpaired *t* test.

Twelve weeks after 20 G needle puncture, no degenerative effect could be determined in C6^+/−^ and C6^−/−^ rabbit IVDs based on T2 quantification in the sagittal and coronal planes (sagittal: in median 0.98-fold (C6^+/−^) and 0.99-fold (C6^−/−^) T2 value relative to untreated control IVDs; coronal: 1.05-fold (C6^+/−^) and 0.98-fold (C6^−/−^) T2 value relative to untreated control; Figure 4A,B). In the case of EP drilling, T2 values of IVDs determined in the sagittal plane with a 0.93-fold median of untreated controls were not reduced in C6^+/−^ rabbits 12 weeks after treatment (Figure 4C). In C6^−/−^ rabbits, however, the reduction in T2 values after EP drilling (by trend) was greater, with a 0.72-fold median value of respective untreated controls (Figure 4C). In the coronal plane, reductions in the relative T2 value of 0.77-fold for C6^+/−^ and 0.71-fold for C6^−/−^ rabbits were determined in EP-drilled IVDs (Figure 4D). 

Generally, evaluations of relative T2 values revealed that EP drilling partly resulted in a degenerative effect, as demonstrated by the reductions in T2 values. In the sagittal direction, this median effect was more pronounced in C6-deficient rabbits compared to the C6-sufficient group. In contrast, for both genotypes, no clear degenerative effects induced by needle puncture could be observed based on the evaluation of T2 values.

### 3.3. EP Drilling Induces a Lower DHI in C6-Deficient Rabbits Compared to C6-Sufficient Rabbits

Furthermore, the influence of needle puncture or EP drilling on the disc height in C6^+/−^ and C6^−/−^ rabbits was analyzed by determining the DHI based on sagittal X-ray images of the lumbar spines. Thereby, DHIs of needle-punctured or EP-drilled IVDs were normalized to the mean DHIs of untreated control IVDs of the respective IVD level for both genotypes, separately. 

**Figure 5:**
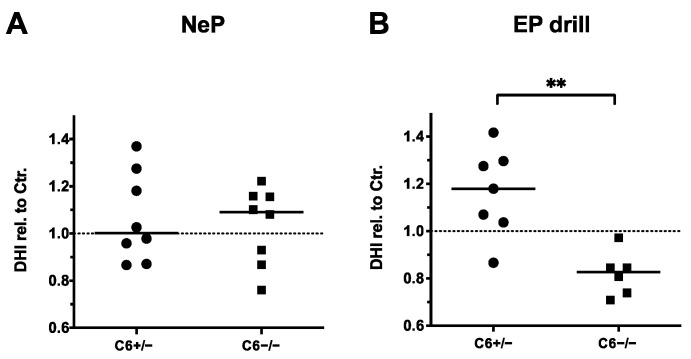
Disc height indexes (DHIs) of treated IVDs relative to untreated controls (dashed lines). DHIs of (**A**) needle-punctured (NeP) and (**B**) EP-drilled (EP drill) lumbar IVDs of C6^+/−^ and C6^−/−^ rabbits normalized to untreated control IVDs of respective IVD level and genotype. Values were determined based on sagittal X-ray images of explanted lumbar spines. DHIs are presented as scatter dot plot with line at median (EP drilled: *n* = 6–7, NeP: *n* = 8 per group). Significant differences are depicted as ** *p* < 0.01; unpaired *t* test.

In accordance with the observations made by MRI-based quantification, 20 G needle puncture did not induce a noticeable reduction in the disc height in C6^+/−^ and C6^−/−^ rabbits (Figure 5A). By determining the relative DHIs of EP-drilled IVDs, no degenerative effects were observed in C6^+/−^ rabbits (relative DHI in median: 1.18, Figure 5B). However, with a median value of 0.88, the relative DHIs of EP-drilled IVDs were significantly lower in C6^−/−^ rabbits compared to C6^+/−^ animals (*p* < 0.01).

In conclusion, DHI analyses in C6-deficient rabbits indicated a trend of IVD degeneration induced by EP drilling. Therefore, evaluation of DHIs confirmed the previously observed median trend measured by T2 quantification and the degenerative effect observed by MRI-based Pfirrmann grading, but only for C6^−/−^ rabbits. This is in line with the observation that partly degenerative effects of EP drilling were more pronounced in IVDs of C6-deficient rabbits compared to C6-sufficient rabbits when evaluating T2 values in the sagittal plane (Figure 4C). In the present animal model, similar to the MRI-based analyses, no degenerative effect of 20 G needle puncture was observed with DHI analyses.

## 4. Discussion

Immuno-histologic analysis of degenerated human IVDs [13,14] and in vitro analysis using human AF cells [15] previously indicated a contribution of terminal complement activation in DD progression. Consequently, appropriate in vivo studies are needed to provide further insights into the functional role of complement activation products in DD pathomechanisms.

For this purpose, the method of EP drilling, originally established in a porcine model [23], was transferred to rabbits for the first time and was studied in parallel with an established method of needle puncture, which is known to induce mild DD in NZW rabbits [27]. The degenerative effects following EP drilling and needle puncture in C6-deficient rabbits were assessed using X-ray and MRI-based analyses. In particular, it has to be mentioned that the use of a high-resolution 11.7 T MRI scanner allowed us to conduct a remarkably precise visualization of rabbit IVDs, representing a significant improvement compared to previous DD in vivo studies in rabbits using MRI analyses of a significantly lower resolution [35,36].

Comparing both surgical interventions in lumbar IVDs of adult rabbits, we observed a stronger degenerative effect induced by EP drilling compared to 20 G needle puncture in the MRI-based analysis of Pfirrmann grades. In line with the observed trend, this could be also observed in the evaluation of the MRI-based determination of T2 values. Assessment of a DHI based on X-ray analysis confirmed this trend only for C6-deficient rabbits. Relevant pathophysiological consequences of vertebral EP trauma on DD-associated cell-biologic processes were previously described in a rabbit ex vivo model [37,38]. Furthermore, our results confirmed the recently reviewed structural and functional relevance of the cartilaginous EP, which has been shown to play a key role in the early stages of IVD degeneration [7]. 

In the case of untreated control IVDs and needle-punctured IVDs, no morphological differences were observed in the IVDs, irrespective of the C6 genotype. However, based on radiologic DHI analyses, IVDs of C6-deficient rabbits revealed even stronger degenerative effects induced by EP drilling compared to identically treated IVDs of C6-sufficient animals. Furthermore, residuals of the drilling channel were visible in MRI scans 12 weeks after surgical intervention about four times more often in C6-deficient animals, possibly indicating impaired bone regeneration. 

Similar findings were previously reported for a C6 deficiency in the study of Mödinger et al., who observed a negative effect of a C6 deficiency on bone fracture healing in mice [39]. The pathophysiological processes triggered by EP drilling exhibit some similarities to the respective fracture healing processes. Therefore, our present observations support the assumption that bone healing may be impaired by a C6 deficiency.

In an anterior cruciate ligament transection (ACLT) model, previously applied in the same rabbit breeding program for the induction of osteoarthritis (OA), an overall protective influence of a C6 deficiency regarding OA development was observed, although an investigation of the subchondral bone indicated compromised bone remodeling in C6-deficent rabbits [25]. Thus, TCC formation may have a somehow divergent influence in skeletal trauma models, depending on the trauma-induced aspects that are analyzed.

Based on the present results of Pfirrmann grading, T2 analysis, and the investigation of the DHIs of lumbar rabbit IVDs, there is no clear evidence of a central functional role of TCC formation in trauma-induced DD in rabbits. Moreover, DHI analyses of EP-drilled IVDs suggest that a certain amount of TCC formation might even have a supporting effect on the maintenance of disc height. However, it has to be considered that despite the lack of functional C6 resulting in the inability of TCC formation, anaphylatoxins C3a and C5a, which strongly contribute to inflammation by immune cell recruitment and cytokine induction [40,41], can be formed normally upon a complementary activation in C6-deficient conditions, and thus could mount any classical sign of inflammation. Therefore, the role of anaphylatoxins in DD development should be analyzed in future studies.

In contrast to the reported findings of previous studies [27,28], we could not observe significant degenerative effects in rabbit IVDs induced by needle puncture. In the present study, surgical intervention techniques were performed in a minimally invasive procedure to reduce the traumatization of surrounding tissue and the potential risk of an infection. The intervention could be applied quite precisely and with the lowest possible unintended additional traumatic lesion. Possibly, this may have contributed to the absence of clear degenerative effects. Furthermore, according to O’Connell et al., lumbar IVDs of adult female New Zealand white rabbits (NZW) have a mean disc height of 1.42 ± 0.39 mm [42], whereas lumbar IVDs of the rabbit strain used for the present study exhibited disc heights of 1.94 ± 0.29 mm (C6^+/−^, *n* = 7), respectively 1.85 ± 0.36 mm (C6^−/−^, *n* = 8; median ±SD). Therefore, the 20 G needle used in this study with a diameter of 0.91 mm covered approximately 48% of the disc height. This ratio allowed us to carry out a precise application of the needle into the central area of the IVD without scratching and damaging the adjacent EP. In other studies, the needle puncture was applied in adult NZW rabbits using needles with a diameter of 1.27 mm (18 G) or even 1.65 mm (16 G) [27,28]. Considering that the mean disc height of lumbar IVDs of the used NZW rabbits are smaller than the height measured for rabbits of the present study [41], it can be concluded that this resulted in a significantly larger ratio of needle diameter to disc height. In the study of Masuda et al., the application of needle puncture using a 21 G needle (0.82 mm diameter) also led to less-pronounced degenerative effects compared to 16 G and 18 G needles [28]. 

Therefore, we conclude that achieving significant DD development is strongly dependent on the diameter of the used needle, with the needle size having to be carefully selected and evaluated depending on the IVD height of the used rabbit strain. Consequently, needles with smaller diameters (below a certain proportional limit value—in relation to the IVD height) do not cause any significant degenerative effects of the IVD. This suggestion agrees with the previous recommendation that smaller needles may be used to introduce growth factors or pharmaceutical reagents without causing damage to tissues [28,43]. Therefore, the larger lumbar disc height of our rabbits might have contributed to the absence of major degenerative effects of IVD puncture using 20 G needle puncture.

Besides disc height, the overall genetic background of the rabbits used in this study representing a chinchilla bastard strain [25] may have influenced the progression of DD. Therefore, the effects of needle puncture may be somehow different compared with the NZW rabbits used in previous studies [27,28]. Furthermore, a residual influence of the genetic background of heterozygous C6^+/−^ rabbits used as a control on the development of DD induced by EP drilling or needle puncture cannot be completely excluded. However, this is rather unlikely, since heterozygous C6^+/−^ rabbit serum exhibits a comparable erythrocytes lysis capacity to NZW serum; moreover, we could show that a respective substitution with human C6 protein could completely restore the erythrocyte lysis capacity of serum from homozygous C6^−/−^ rabbits (Supplementary Figure S1). Nevertheless, further validation of the EP drilling method in a commonly used strain like NZW rabbits is necessary in the future. Furthermore, it should be kept in mind, that by considering the principles of 3R, this first study could in part only reveal tendencies. For more detailed insights, larger group sizes should be considered. 

Moreover, previous in vivo studies assessing X-ray-determined DHIs as well as MRI-based T2 quantifications indicated that the degenerative effects of surgical interventions on rabbit IVDs were increased in a time-dependent manner [44]. Therefore, we propose to analyze the development of DD induced by EP drilling in the course of time in future studies.

Considering the surgical intervention applied to induce DD, the method of EP drilling according to Holm et al. more closely mimics the conditions of trauma-induced IVD degeneration based on EP injuries in humans—in contrast to other surgical interventions applied in vivo—as it induces degeneration of the tissue, which develops evenly distributed over the whole IVD [23]. Although the surgical procedure is quite challenging and was performed by a team of trauma and spine surgeons in the present study, minimally invasive EP drilling seems to be a suitable method regarding lumbar rabbit IVDs for the induction of DD. Compared to domestic pigs, the application of EP drilling in rabbits offers the advantage of a less cost-intensive animal model. In rabbits, due to the anatomic proximity of the lowest ribs, accurate positioning of the drill at level L1/2 proved to be technically difficult and not successful. Therefore, level L1/2 should be excluded from EP drilling of lumbar rabbit IVDs. Furthermore, we propose to perform the method of EP drilling on lumbar rabbit IVDs under a fluoroscopic control [45] to further optimize visualization and facilitate technical implementation.

Overall, the limitations of this study are as follows: a low sample size, the analysis of only one time point, no continuous imaging control during surgery, and a lack of a histomorphological evaluation.

## 5. Conclusions

Based on the present results of Pfirrmann grading, T2 analysis, and the investigation of the DHI of lumbar rabbit IVDs, there is no clear evidence of a central functional role of TCC in trauma-induced DD in rabbits. This suggests that the disease severity-associated TCC deposition in the intervertebral discs of DD patients might be primarily regarded as a marker of local complementary activation. Therefore, the functional implication of other complementary activation products, especially anaphylatoxins—which are not affected by a C6 deficiency—should be examined more closely in further studies. In this study, we could show that minimally invasive EP drilling represents a challenging but appropriate method for inducing degenerative effects on lumbar rabbit IVDs.

## Figures and Tables

**Table 1:** Scheme of surgical treatments groups A–D in alternating arrangement of treatment and control IVDs. EP drill: endplate drilling, NeP: needle puncture, Ctr.: untreated control IVD.

	A	B	C	D
**L1/2**	EP drill	Ctr.	NeP	Ctr.
**L2/3**	Ctr.	EP drill	Ctr.	NeP
**L3/4**	NeP	Ctr.	EP drill	Ctr.
**L4/5**	Ctr.	NeP	Ctr.	EP drill

## Data Availability

The data presented in this study are available upon request from the corresponding author.

## Supplementary Materials

The following supporting information can be downloaded at: https://www.mdpi.com/article/10.3390/biomedicines12081692/s1, Figure S1: Genotyping of animals from C6-deficent rabbit breeding; Table S1: Gender, age, and weight of individual rabbits of both genotypes (C6^+/−^ and C6^−/−^) at the time point of surgery; Figure S2: Post-operative weight monitoring of individual C6^+/−^ and C6^−/−^ rabbits. References [30,46] are cited in the Supplementary Materials.
