# Supplementary material for: Analysis of Intervertebral Disc Degeneration Induced by Endplate Drilling or Needle Puncture in Complement C6-Sufficient and C6-Deficient Rabbits
Source: Biomedicines. 2024 Jul 30;12(8):1692. doi: 10.3390/biomedicines12081692 (PMC11351780; doi:10.3390/biomedicines12081692)
Supplement: Supplementary file 1 [file biomedicines-12-01692-s001.zip › biomedicines-3113318-supplementary.pdf]

Genotyping of animals from C6-deficent rabbit breeding

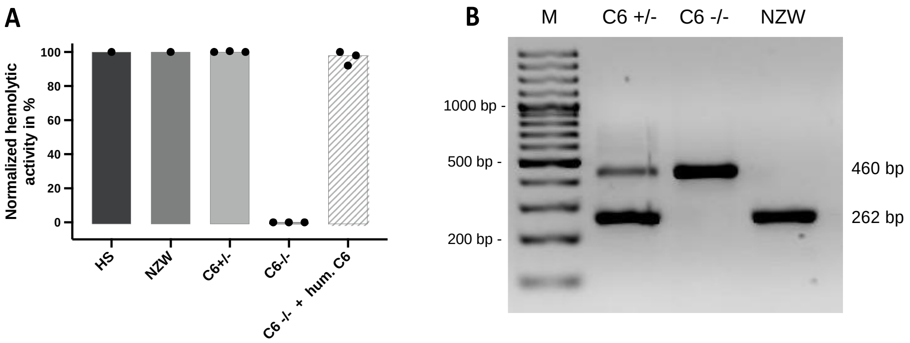

Figure S1. Genotyping of animals from C6-deficent rabbit breeding. (A) Analysis of hemolytic activity of rabbit serum from wild-type rabbits (New Zealand white rabbits, NZW), C6-sufficient ($C6^{+/-}$), and C6-deficient ($C6^{-/-}$) rabbits of the breeding using sensitized sheep erythrocytes. As reference human serum (HS) was analyzed. Lytic activity was normalized to a complete lysis control. While serum of $C6^{+/-}$ rabbits exhibit a hemolytic activity comparable to NZW serum and HS, hemolytic activity of $C6^{-/-}$ serum was nearly completely lost. Supplementation of $C6^{-/-}$ rabbit serum with human C6 protein (hum. C6; 14 µg/mL, correspondent to the C6 content in $C6^{+/-}$ rabbit serum [46]) could completely restore the hemolytic activity to the level of NZW serum. This demonstrates that the lack of complement activity is limited to the C6 deficiency only. (B) In further analysis, DNA of the rabbits were analyzed by means of a PCR amplifying sequences of the *C6* gene. Thereby amplicons resulting from wild-type *C6 ($C6^{+}$)* had a size of 262 bp, whereas the mutated *C6* gene *($C6^{-}$)* was template for amplicons of 460 bp length.

Additional information about housing/ health monitoring

The rabbits were derived from in-house breeding originally descending from a C6-deficient rabbit [30]. Detailed information to individual rabbits used in this study are given in Table S1. From an age of 10 weeks onwards, they were hold in single cages in agreement with the official European Guideline 2010/63/EU.

Hygiene status was monitored according to the official FELASA criteria. The weight monitoring (Figure S2) as well as the daily wound and behavior controls did not show alarming anomalies.

Table S1. Gender, age and weight of individual rabbits of both genotypes ($C6^{+/-}$ and $C6^{-/-}$) at the time point of surgery. Gender: m – male, f – female.

$C6^{+/-}$	Gender	Age in weeks at surgery	Weight at OP (in kg)
Animal 1	m	41	4.05
Animal 2	f	41	4.64
Animal 3	m	41	3.35
Animal 4	m	41	3.96
Animal 5	f	39	3.54
Animal 6	f	39	3.96
Animal 7	f	39	3.93
Animal 8	m	35	3.58
Animal 9	m	41	3.91
**$C6^{-/-}$**	**Gender**	**Age in weeks at surgery**	**Weight at OP (in kg)**
Animal 1	m	41	3.82
Animal 2	m	39	3.52
Animal 3	f	39	3.85
Animal 4	f	39	3.85
Animal 5	f	39	3.56
Animal 6	m	39	3.66
Animal 7	f	39	3.67
Animal 8	m	35	3.26

Post-operative weight monitoring of individual C6$^{+/-}$ and C6$^{-/-}$ rabbits.

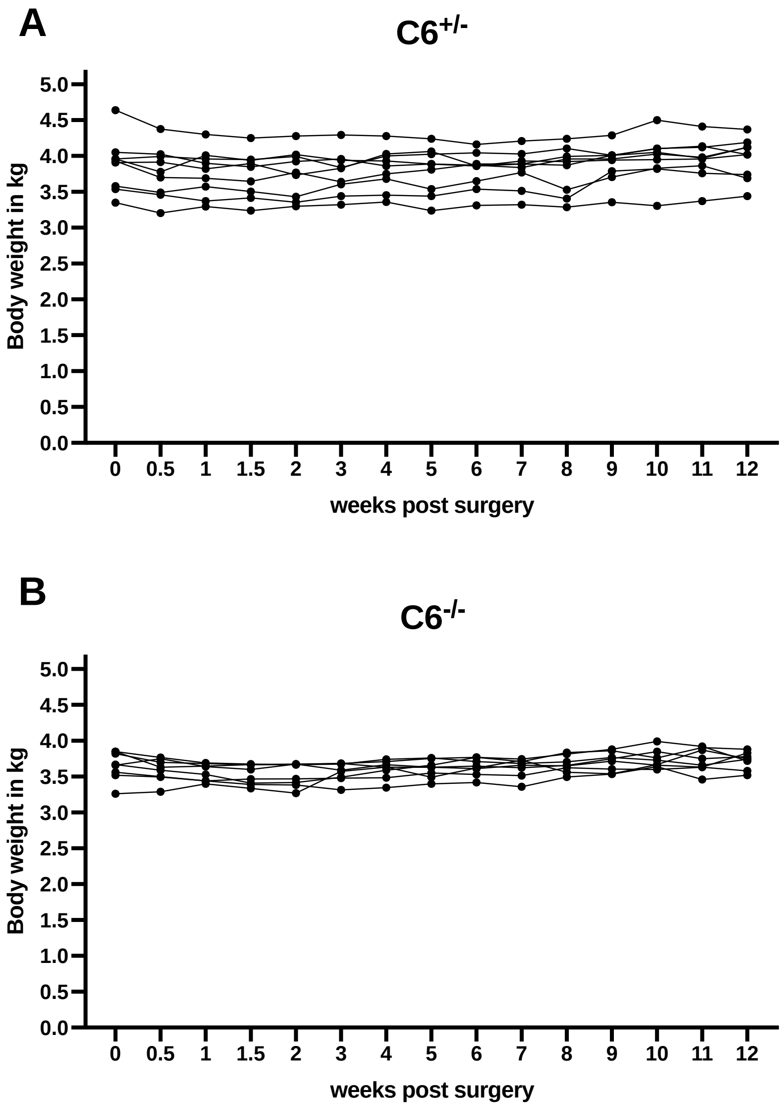

Figure S2. Post-operative weight monitoring of individual C6$^{+/-}$ (A) and C6$^{-/-}$ (B) rabbits. Body weight in kg of each animal monitored from pre-surgery (week 0) until harvest 12 weeks post-surgery. Data points related to the same animal are connected with lines. No significant weight loss was observed.
